# THE ROLE OF APPRAISAL IN CAREGIVER CHARACTERISTICS ASSOCIATED WITH ELDER NEGLECT

**DOI:** 10.1093/geroni/igae098.0722

**Published:** 2024-12-31

**Authors:** Eleanor Batista-Malat, Zachary Gassoumis, Kathleen Wilber, Bonnie Olsen, Laura Mosqueda

**Affiliations:** University of Southern California, Los Angeles, California, United States; University of Southern California, Los Angeles, California, United States; University of Southern California, Los Angeles, California, United States; University of Southern California, Los Angeles, California, United States; University of Southern California Keck School of Medicine, Pasadena, California, United States

## Abstract

Elder neglect occurs when a caregiver fails to meet a need, compared to elder abuse which involves specific abusive actions. Despite this, neglect and abuse are typically studied together as elder mistreatment. We investigated the characteristics associated with neglectful behaviors of caregivers of people living with dementia (PLWD). California dementia caregivers (N=92) completed a cross-sectional anonymous online survey about their caregiving experience and mistreatment of the PLWD from any source. 44.6% of caregivers were caring for a parent, 51.1% were Hispanic/Latino, and 68.1% were female. Eighteen (19.6%) caregivers reported at least one neglectful behavior in the last year, defined as leaving the PLWD alone inappropriately, not providing them with needed medical care, food, or water, or being impaired in caregiving due to alcohol or drugs. Of these, 11 (61.1%) also reported abuse in the last year: nine psychological, five financial, and five physical abuse. Bivariate tests comparing characteristics of caregivers with and without neglectful behaviors showed no difference in time spent caregiving, outside caregiving help, or social support and loneliness. Those who reported neglectful behaviors were significantly more likely to live with the PLWD (X2 =7.2, p=.028), believe the PLWD could “do better if they tried” (X2 =9.0, p=.029), and have higher burden scores (t=-2.61, p=.011). Our findings suggest that caregiver neglect may be associated with the caregivers’ perceived experiences rather than objective measures. Service providers should be attentive to caregivers’ understandings of their experiences, which may be related to neglectful behaviors, and reducing neglectful behavior may reduce overall mistreatment.

